# Sleep and Speech Outcomes After Superior Adenoidectomy in Children with Cleft Palate

**DOI:** 10.7759/cureus.2097

**Published:** 2018-01-21

**Authors:** Emily Waselchuk, James D Sidman, Timothy Lander, Robert Tibesar, Brianne B Roby

**Affiliations:** 1 Otolaryngology, University of Minnesota; 2 Otolaryngology Head and Neck Surgery, University of Minnesota; 3 Pediatric Ent and Facial Plastic Surgery, Children's Hospital of Minnesota

**Keywords:** cleft palate, craniofacial, sleep disordered breathing, adenoidectomy, nasal obstruction, obstructive sleep apnea

## Abstract

Objective

To describe the sleep and speech outcomes in patients with cleft palate who underwent superior adenoidectomy.

Subjectives and methods

This is a case series with chart review of patients with diagnoses of cleft palate and sleep disordered breathing (SDB), obstructive sleep apnea (OSA) or nasal obstruction treated with superior adenoidectomy from 1991-2015 at the Children’s Hospital of Minnesota. Postoperative clinic notes documented the changes in symptoms following surgery. All speech outcomes were recorded.

Results

Fifty patients (23 females, 27 males) aged 11 months to 17 years were included. Forty-six patients (92%) had improvement of sleep symptoms including snoring, nighttime restlessness, and witnessed apnea events, following superior adenoidectomy. Forty-two of the 46 patients (91%) had stable speech postoperatively with either no development or no worsening of velopharyngeal insufficiency (VPI).

Conclusion

Superior adenoidectomy is an effective procedure to alleviate symptoms of sleep disordered breathing in patients with cleft palate without significantly affecting speech.

## Introduction

Cleft palate is a common birth defect in the pediatric population, with an incidence of approximately one in 680 live births [1]. Resulting from the failure of the medial nasal process to maintain contact with the lateral nasal and maxillary processes, cleft palates affect speech, middle ear function, and are suspected to significantly contribute to airway obstruction and sleep disordered breathing. Airway obstruction in patients with craniofacial abnormalities, including cleft palate, is multifactorial. Issues with nasal vestibule malformations, septal deviation, midface deformities, and anatomical changes associated with procedures like pharyngeal flap and sphincter pharyngoplasty can all cause airway obstruction [2]. Several studies have shown that the incidence of sleep disordered breathing (SDB) and obstructive sleep apnea (OSA) is between 22-37%, as compared to less than 5% of the non-cleft pediatric population [1]. Obstructive sleep apnea is characterized by snoring, labored breathing while asleep, daytime sleepiness, restlessness, and even learning and behavioral problems. Because of these unsettling results of OSA, surgical treatment, most specifically adenoidectomy and tonsillectomy, is a first line treatment to alleviate the obstruction and improve sleep [3]. The problem with adenoidectomy in the cleft population is the increased risk of creating or worsening velopharyngeal insufficiency (VPI) and altering the patient’s speech. Though adenoidectomy is one of the most common procedures performed in children, the risk of VPI is well known and is inherently higher in children with cleft palate. To avoid hypernasal speech, nasal emission, and nasal regurgitation, which are characteristics of VPI, several methods of performing partial adenoidectomy have been described. Most techniques involve removal of adenoid tissue obstructing the posterior choanae and leaving residual adenoid tissue inferiorly. The residual adenoid tissue is left intentionally to ensure there is contact during palatal elevation [3]. The surgery is typically performed under indirect vision with a mirror and with a monopolar cautery device, suction shaver or a coblator. The Children’s Hospital of Minnesota has a large census of cleft palate patients, and superior adenoidectomy is routinely performed to correct sleep disordered breathing and avoid VPI. This study reports the speech and sleep outcomes in patients with cleft palate after superior adenoidectomy.

## Materials and methods

This study is a case series with chart review examining patients with a diagnosis of cleft palate who underwent superior adenoidectomy for the treatment of sleep disordered breathing or obstructive sleep apnea between 1991 and 2015 at the Children’s Hospital of Minnesota. Patients were identified using Current Procedural Terminology (CPT) codes for “adenoidectomy” and cross-referenced to patients with International Classification of Diseases, Ninth Revision (ICD-9) codes of “cleft palate,” “obstructive sleep apnea”, “sleep disordered breathing”, and “nasal obstruction.” Patients were included in this study if they had a history of either a cleft palate or a submucous cleft palate, if they had a diagnosis of sleep disordered breathing or obstructive sleep apnea, and if they underwent superior adenoidectomy. The children with overt cleft palate had previously undergone palatal repair. Patients who underwent tonsillectomy with superior adenoidectomy concurrently were excluded from the study as this study aimed to evaluate superior adenoidectomy alone. However, patients with a previous tonsillectomy and persistent evidence of nasal obstruction with sleep disordered breathing were included. Patients were also excluded from the study if they had a history of speech surgery. The surgery was performed by four attending surgeons. In some cases, residents and fellows assisted with the surgery, however, the amount of residual adenoid tissue was always confirmed by the attending surgeon.

All patients were evaluated by a multidisciplinary cleft palate and craniofacial team including a speech language pathologist and pediatric otolaryngologist. Preoperative clinic notes documented a perceptual speech assessment as well as symptoms of sleep disordered breathing including snoring, nighttime restlessness, and witnessed apneic events.

Postoperative evaluation took place within eight weeks of surgery. Sleep symptoms and speech assessment were also documented. One of several speech therapists who were experts in cleft and craniofacial velopharyngeal insufficiency performed the speech analysis.

This study was approved by the Institutional Review Board at the Children’s Hospital of Minnesota.

Surgical technique

After induction of general anesthesia, patients were intubated and positioned supine with shoulder roll in place for neck extension. A McIvor gag was placed and suction catheters were passed transnasally to retract the soft palate and expose the adenoid bed and choanae. The nasopharynx and adenoid pad were visualized under indirect vision using a laryngeal mirror. Adenoids were then removed using either a suction microdebrider (Straightshot™ M4 Microdebrider, Medtronic, Inc, Jacksonville, FL ​​​​​​) or a malleable suction coagulator (Valleylab™, Covidien, Dublin, Ireland). Tissue removal was carried out in the area limited to the posterior choanae. No adenoid tissue within 7 mm of Passavant’s ridge was removed. Care was taken to avoid injury to the eustachian tube orifices, septum, and inferior turbinates. The adenoid bed was packed with tonsil sponges and removed after 60 seconds. Final hemostasis was achieved with suction cautery.

## Results

**Figure 1:**
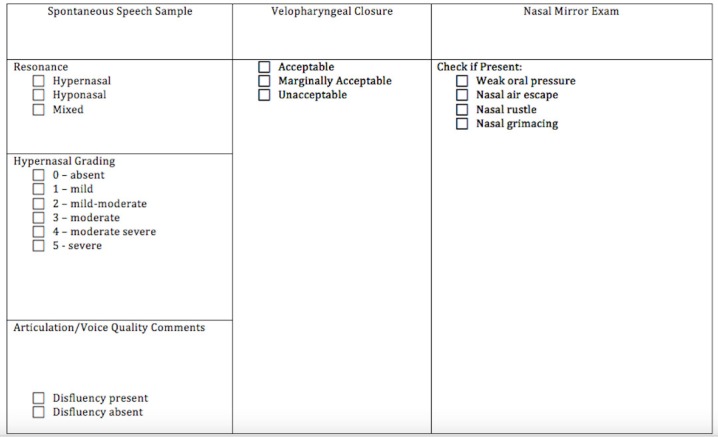
Perceptual Speech Assessment Form

Between 1991 and 2015, 50 children with cleft palate underwent superior adenoidectomy for the relief of sleep disordered breathing. There were 23 female and 27 male patients. The patients’ ages ranged from 11 months to 17 years. A full list of patient demographics is shown in Table 1. Ten patients had mild to moderate velopharyngeal insufficiency, as graded by the speech therapist, preoperatively. Forty-six patients (92%) had improvement of sleep symptoms including snoring, nighttime restlessness, and witnessed apneic events, following superior adenoidectomy. The four remaining patients had no improvement, but no worsening in sleep symptoms. All 50 patients had documented postoperative speech evaluations between six and eight weeks following surgery. The speech evaluations were performed by a professional holding a Certificate of Clinical Competence in Speech Language Pathology (CCC-SLP) who works specifically with the cleft and craniofacial team, specializing in evaluating and treating children with cleft palate. Speech evaluations were systematic and included perceptual assessment of resonance including analysis of speech and/or oral reading when appropriate. Nares occlusion was used to assess for a shift of resonance or improvement in oral pressure in production of words. Nasal mirror testing was utilized to detect nasal air escape. Resonance was described as hypernasal, hyponasal, or mixed. Speech assessments also included screening of articulation skills and voice quality. Based on speech and resonance characteristics, velopharyngeal closure was judged to be acceptable, marginally acceptable, or unacceptable. Figure 1 demonstrates a sample of an assessment form. Four patients were non-verbal and speech could not be assessed. The remaining 46 patients had stable speech postoperatively with either no development or worsening of VPI. After the initial postoperative visit, the range of follow-up spanned from two months to 10 years. Only two patients had symptomatic adenoid regrowth requiring re-excision. None of the patients in this study required speech surgery.

**Table 1:** Patient Demographics

Age	11 mo - 17 yrs (avg 3.58 yrs)	
Sex	Male	27
	Female	23
Pre-op Diagnosis	Submucous Cleft Palate	14
	Incomplete Cleft Palate	3
	Complete Cleft Palate	22
	Complete Cleft Lip and Palate	10
	Bilateral Complete Lip and Palate	1
Surgical Technique	Suction Cautery	21
	Microdebrider	25
	Not Specified	4

## Discussion

The prevalence of sleep disordered breathing in children with cleft palate is roughly three times higher than in children without cleft palate. The majority of children present with sleep symptoms following their cleft repair regardless of age [1]. The most common procedure performed for patients with sleep disordered breathing is a combination tonsillectomy and adenoidectomy. In this study all patients evaluated had a superior adenoidectomy alone. After adenoidectomy, the degree of palatal closure can be disrupted and result in VPI. Although uncommon in the general pediatric population, aggressive adenoidectomy can decrease the quality of speech significantly in patients with cleft palate. These children have a short palate and typically have palatal dysfunction [4]. Because the long-term sequelae of SDB can be debilitating from a behavioral standpoint and dangerous from a cardiopulmonary standpoint, surgical intervention is generally necessary even though adenoidectomy has classically been approached with caution in patients with cleft palate [5]. Superior adenoidectomy is indicated in these cases to preserve the patient’s speech while removing the bulk of the choanal obstruction.

The data in this study demonstrates that superior adenoidectomy is effective and does not have any adverse effects on speech while improving symptoms of sleep disordered breathing. This subset of patients demonstrated classic symptoms of sleep apnea with associated physical exam findings and thus sleep studies were not performed as it was felt a sleep study would not change surgical management. Pre- and post-op polysomnography (PSG) data would provide objective evidence to demonstrate improvement in sleep following surgery and would be useful to include in future studies. The American Academy of Otolaryngology - Head and Neck Surgery (AAO-HNS) practice guidelines recommend PSG prior to tonsillectomy in children with sleep disordered breathing who have craniofacial abnormalities such as cleft palate; however, the patients in this study underwent adenoidectomy alone and thus a preoperative PSG was not indicated. Patients with tonsillectomy were not included in this study as the aim was to investigate the efficacy of superior adenoidectomy alone. There have been studies showing comparable subjective outcomes measured by the Pediatric Sleep Questionnaire (PSQ) in children with small tonsils who underwent adenoidectomy alone rather than adenotonsillectomy [6]. Of note, this case review includes patients from as early as 1991, which is before guidelines about sleep study in craniofacial patients was established and also before the PSQ was developed. Several recent studies have shown that PSQ is not the most reliable tool for patients with craniofacial disorders [7]. 

Because the rate of sleep disordered breathing and baseline VPI is significantly higher in children with cleft palate, appropriate surgical technique is necessary to cure the obstruction and preserve speech quality. Superior adenoidectomy improves sleep outcomes by removing a limited amount of adenoid tissue at the choanae, while preserving enough tissue for adequate palatal closure.

## Conclusions

Sleep disordered breathing and obstructive sleep apnea are common problems in patients with cleft palate. Surgery is indicated to remove the obstruction, but it is not without risk. Aggressive adenoidectomy has the potential to negatively affect speech. This case series with chart review of a large cohort of pediatric cleft palate patients demonstrates that performing superior adenoidectomy in children with cleft palate is an effective method to improve sleep symptoms without producing or worsening VPI.
